# Genital Lichen Sclerosus: From Hidden Mechanisms to Healing Horizons

**DOI:** 10.3390/ijms27198807

**Published:** 2026-10-01

**Authors:** Angela Filoni, Noemi Aprile, Silvia Caputo, Barbara Bellei

**Affiliations:** 1 UOSVD Dermatology and Allergology, Hospital Vito Fazzi, 73100 Lecce, Italy; noemi.aprile07@hotmail.it; 2 Laboratory of Cutaneous Physiopathology and Integrated Center of Metabolomics Research, San Gallicano Dermatological Institute, IRCCS, 00144 Rome, Italy; silvia.caputo@ifo.it

**Keywords:** lichen sclerosus, fibrosis, inflammation, dermatosis

## Abstract

Lichen sclerosus (LS) is a chronic inflammatory dermatosis that primarily affects the genital mucosa in both sexes. Although most prevalent in women and characterized by distinct age peaks, its clinical presentation, marked by ivory-white atrophic patches and plaques, often overlaps with other conditions. Delayed diagnosis, frequently due to asymptomatic progression, poses a significant risk of malignant transformation. The etiology of LS is multifactorial, involving a complex interplay of genetic, epigenetic, environmental, and autoimmune factors. Pathogenetically, the disease is characterized by several co-occurring features, including an immune imbalance with elevated pro-inflammatory cytokines, impaired T-regulatory (Treg) cell activity, a persistent local inflammatory state, and dysregulated collagen metabolism with associated fibrosis and scarring. Furthermore, the production of autoantibodies against extracellular matrix protein 1 (ECM1) disrupts skin integrity, while the generation of reactive oxygen species (ROS) further exacerbates tissue damage, autoimmunity, and tumorigenesis. Despite its severe impact on patient quality of life, current diagnostic and therapeutic strategies remain limited. While potent topical corticosteroids represent the gold standard first-line treatment—with topical calcineurin inhibitors serving as a second-line alternative—the lack of a fully elucidated etiopathogenesis continues to restrict curative options. However, recent translational research is paving the way for innovative regenerative therapies. This review provides an updated comprehensive overview of the latest advancements in LS pathogenesis and explores emerging therapeutic landscapes.

## 1. Introduction

Lichen sclerosus (LS) is a mucocutaneous chronic inflammatory disease of unknown etiology, marked by a progressive, recurrent nature, involving the perineal and external genital regions (85–98%), while the extragenital involvement is less common (15–20%) and may affect areas such as the face, neck, shoulders, trunk, and back [1]. In the early phase, LS manifests with depigmented, atrophic patches that progressively evolve into plaques. Initially, hyperkeratosis, blistering, and ulceration occur sporadically, and patients are often minimally symptomatic. Advanced stages involve profound remodeling of tissue architecture, leading to atrophy, sclerosis, ulceration, and destructive scarring. In women, this may cause clitoral phimosis and labial adhesions, whereas in men, the predominant features include frenulum breve, phimosis, foreskin adhesions to the glans, and meatal stenosis [2]. Clinically, disease progression manifests as pruritus, itching, burning and pain [3]. Therefore, diagnosis often arises when the disease is in a late stage based on clinical evaluation and sometimes without histological evaluation [4]. LS is also associated with urinary and sexual dysfunction, with considerable impact on the quality of life of affected patients [5,6]. The course of LS is mostly chronic, with serious implications for skin cancer risk [7,8].

The prevalence of LS is often underestimated due to the psychological aspect, misdiagnosis if dermatologists are not involved, and sometimes the lack of symptoms in the early stages [4]. Additionally, its clinical features can overlap with diseases such as lichen planus (LP), vitiligo, eczema, psoriasis, autoimmune blistering diseases and candidiasis [1,9]. LS can occur at every age and in both sexes, with a male-to-female ratio between 1:3 and 1:10 [2,10]. In women, LS exhibits a bimodal incidence with peaks in prepubertal girls and postmenopausal women [11]. In contrast, male LS demonstrates a triphasic distribution: it presents during childhood and follows two distinct peaks in adulthood—the first at the end of the third decade and the second during the sixth decade of life [12].

LS is a multifactorial disease involving complex interactions among genetic predisposition (associated with HLA class II antigens, particularly HLA-DQ7), epigenetic alterations and environmental factors. These factors elicit an aberrant immune response characterized by a dysregulation of pro-inflammatory cytokines and T-regulatory (Treg) cell dysfunction. This immunological imbalance is considered a possible driver for collagen metabolism disruption, culminating in extensive fibrosis and cicatricial scarring of the affected tissues [13]. In line with peculiar age-related peaks, hormonal changes are also implicated [14,15].

Accumulating evidence suggests that autoimmunity plays a pivotal role in the pathogenesis of LS. This is underscored by its strong association with other autoimmune conditions, such as thyroiditis, vitiligo, and alopecia areata, which indicates a shared genetic susceptibility, as well as by the presence of specific circulating autoantibodies [9]. Consequently, this narrative review aims to provide a comprehensive update on the evolving landscape of LS pathogenesis and current therapeutic strategies.

## 2. Materials and Methods

This narrative review did not apply strict systematic exclusion criteria prior to retrieving the electronic literature. A comprehensive search was conducted across the PubMed and Scopus databases for studies published up to August 2026. Keywords used included combinations of terms such as “lichen sclerosus”, “genital lichen sclerosus”, “vulvar lichen sclerosus”, “male genital lichen sclerosus”, “pathogenesis”, and “therapeutics”. Records were screened based on topical relevance, clinical evidence quality, and language (English), prioritizing peer-reviewed original studies and recent high-impact reviews to ensure a thorough synthesis of the current literature.

## 3. Coexisting Autoimmune and Non-Autoimmune Diseases

A systematic review and meta-analysis conducted up to February 2024 [16] demonstrated that both female and male patients with LS had significantly higher odds of common skin conditions, such as lichen planus, vitiligo, alopecia areata, atopic dermatitis and psoriasis, as well as non-dermatological disorders, including hypertension, obesity, dyslipidemia, type 1 and type 2 diabetes and hypothyroidism (including Hashimoto thyroiditis) compared to non-LS controls. More recently, data from an Australian database indicate a high prevalence of comorbid autoimmune diseases, affecting 24.5% of pediatric cases and 34.6% of patients with vulvar LS [17]. The study by Kreuter et al. highlights significant sex-based differences in the association between LS and autoimmune disorders. While both sexes exhibit a link to autoimmunity, the prevalence is substantially higher in women (28%) than in men (10.6%). Female patients were more frequently diagnosed with concomitant autoimmune thyroiditis, localized scleroderma, and vitiligo. Furthermore, the presence of autoantibodies was notably more common in women (40.4%) than in men (18.6%), suggesting that the autoimmune component of LS pathogenesis may be significantly more pronounced in female patients [18]. Females with LS additionally showed significant associations with systemic lupus erythematosus, urinary incontinence, coeliac disease, depression and other thyroid diseases, while males with LS were significantly associated with nongonococcal urethritis and coronary artery disease [16]. The presence of these autoimmune comorbidities suggests a dysfunction in regulatory T-cell-mediated suppression, potentially driving the loss of self-tolerance toward extracellular matrix antigens observed in the pathogenesis of lichen sclerosus [19,20,21,22,23]. Furthermore, cardiovascular risk factors and diseases may exacerbate endothelial dysfunction and decrease microvascular density in LS tissue [24,25]. The coexistence with Morphea, a rare inflammatory disease affecting the skin and connective tissue leading to sclerosis and atrophy, has been sometimes reported for vulvar LS and extragenital LS [26,27,28]. Furthermore, sporadic cases of multiple autoimmune syndromes comprising LS have been described in the literature [29,30].

However, the association with other autoimmune diseases does not necessarily imply a causative factor. Further studies are necessary to correlate the local immune profile and the systemic immune dysregulation.

Genital dysplasia is frequent, and there is also an increased risk of squamous carcinoma of the penis and vulva [31]. The risk of anogenital squamous cell carcinoma (SCC) associated with LS is approximately 2% in men, whereas large epidemiological studies indicate that women with vulvar LS have a lifetime risk of developing SCC ranging from 2% to 7%. On the other hand, up to 65% of vulvar carcinomas arise against a background of genital LS [32,33,34]. Interestingly, in the context of LS, local recurrence of vulvar SCC is more frequent [35]. The association with melanoma and basal cell carcinoma (BCC) is sporadically reported in male and female patients with LS [36,37,38].

## 4. Genetic Predisposition

Genetic predisposition contributes to LS pathogenesis, substantiated by observations of familial aggregation and strong association with Human Leukocyte Antigen (HLA) antigens, particularly with HLA-DQ7. Evidence of hereditary factors emerges from documented familial instances, encompassing monozygotic and dizygotic twin pairs, sibling groups, and mother-to-daughter/son series [39]. A family history of LS has been reported in 5–12% of genital cases across British, Italian, and Dutch cohorts [40].

Studies regarding HLA class II antigens or their haplotypes show significant associations with genital LS versus controls [41,42,43]. A multicenter study on HLA-A/B/DRB1 polymorphisms in the Chinese population demonstrated the association of some genotypes with a higher risk of vulvar LS and of some others with a lower risk of vulvar LS, and a similar trend for squamous cell hyperplasia of the vulva [44]. However, no links have been identified between HLA profiles and factors such as disease initiation, lesion sites, or therapeutic outcomes [43].

Epidemiological studies have also evidenced a link with Turner syndrome, a disorder caused by the complete or partial absence of an X chromosome, which predisposes to LS with an estimated prevalence of 17.3% [45].

Moreover, the above-discussed frequent autoimmune comorbidities argue for intrinsic genetic predisposition to immunological abnormalities.

## 5. Epigenetic Factors

Epigenetic modifications, which change gene expression, also contribute to the pathogenesis of LS. In early stages, hypermethylation of the p16INK4a promoter occurs, driving deregulated cell growth independently of p53 mutation. Hypomethylation appears to be associated with neoplastic transformation in the presence of LS, as this epigenetic modification is significantly more frequent in vulvar SCC co-occurring with LS than in cases unrelated to LS [46]. Epigenetic inactivation of some genes likely favors SCC occurrence since it is also present in lesions adjacent to SCC, but it is absent from SCC-free LS [46]. Unmethylated Thrombospondin (TSP-1) gene has been associated with frequent SCC recurrence, vascular invasion and poor patient outcomes [46]. Changes in isocitrate dehydrogenase enzyme expression led to decreased methylation in the LS-affected epidermis, while ultraviolet A1 (UVA1) therapy normalizes global DNA hypermethylation [47].

## 6. Pathogenic Mechanisms

**Figure 1:**
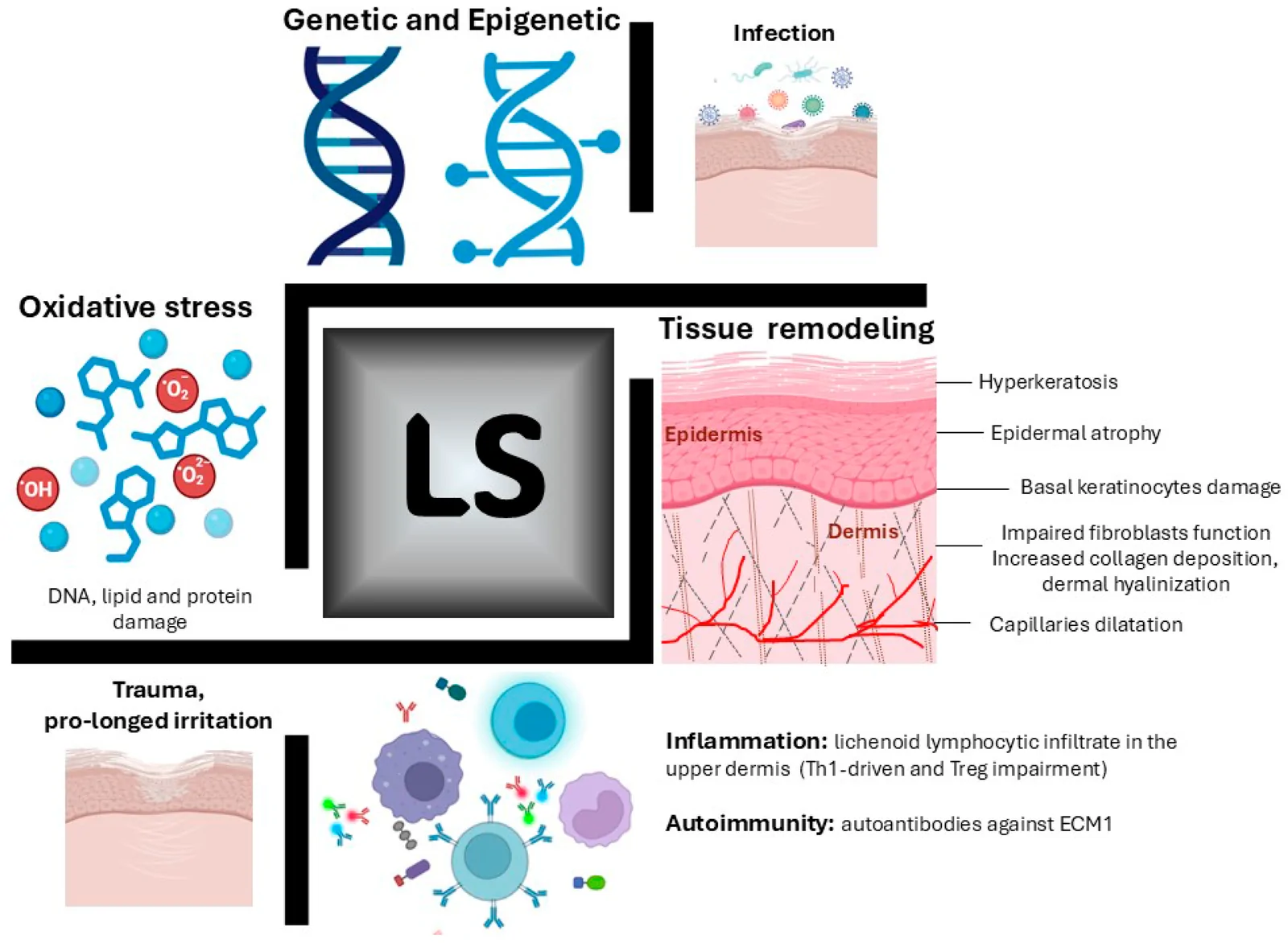
Multifactorial pathogenesis of lichen sclerosus (LS). Schematic overview of the key predisposing triggers, immunological mechanisms, and histopathological alterations in LS. Genetic susceptibility, epigenetic factors, oxidative stress (causing DNA, lipid, and protein damage), infectious agents, and mechanical trauma (Koebner phenomenon) act as potential triggers. These factors contribute to immune dysregulation marked by autoantibodies against ECM1 and a Th1-driven lichenoid lymphocytic infiltrate in the upper dermis with Treg impairment. The resulting tissue remodeling leads to hyperkeratosis, epidermal atrophy, basal keratinocyte damage, altered fibroblast function with increased collagen deposition, dermal hyalinization, and capillary dilatation.

The etiology of LS remains incompletely understood and is the subject of ongoing research. Three primary mechanisms have been proposed to drive its development and progression: (i) persistent inflammation and pathway activation promoting autoimmunity; (ii) deterioration of tissue integrity resulting in characteristic sclerotic and atrophic changes; and (iii) oxidative stress (Figure 1). Additional potential triggers include viral, bacterial, and fungal infections, localized mechanical trauma, chronic urine exposure due to incontinence, and other chemical irritants.

### 6.1. Immunopathogenesis

Humoral and cell-mediated immunity are both implicated in LS [22,48]. B-cells, CD4-positive T cells, antigen-presenting dendritic cells, macrophages and natural killer cells are abundantly detected in LS biopsies [48]. The detection of frequently monoclonally rearranged TCR-gamma on T cells points toward a putative LS-associated antigen. Multiple pro-inflammatory soluble mediators drive disease onset and progression, with intense immune cell infiltration concentrated in the upper-to-mid dermis and along the basement membrane zone, while the lower dermis remains largely spared [49]. These lymphoid cells express mainly the chemokine receptors CXCR3 and CCR5 and lack CCR3 and CCR4, suggesting a Th1 profile. This Th1 response intensifies through the production of interferon γ and the attraction of more Th1 cells. Other pro-inflammatory cytokines such as IL-1α, IL-7, IL-15, and TNF-α, as well as immune mediators such as IL-2 receptor (CD25), caspase 1, ICAM-1, and its ligand CD11a are upregulated in LS [13], whereas anti-inflammatory cytokines (e.g., IL-10) are downregulated [50,51]. Genetic studies have identified an intronic polymorphic variant in the gene encoding the interleukin-1 receptor antagonist (IL-1Ra), a competitive inhibitor of IL-1 alpha and IL-1 beta [52]. Supporting the significance of this endogenous anti-inflammatory molecule, specific allelic variants have been correlated with increased clinical severity of the disease [52]. Czajkowski et al. found that IL-1α, IL-6, and IFN-γ were detectable in early, moderate, and severe stages of penile LS, respectively, with disease progression being closely related to micro-incontinence [53]. This type-1 cytokine cascade is further amplified by the upregulation of chemokine and their receptors, including CXCR3 and its ligands CXCL9-11, as well as CCR5, with CCL4 and CCL5, which promote the recruitment of inflammatory cells into lesional tissue [50,54]. IL-4/IL-13 signaling may also play a role in LS, particularly in cases characterized by pruritus and lichenification. Consistently, Carli et al. [55], comparing vulvar LS with vulvar lichen planus (LP) in women, first reported that in vulvar LS there is increased IL-4 expression (a marker of T helper 2 profile and of a fibrotic response), especially in early lesions, while IFN-γ staining was less than 10% infiltrating cells; an inverse IL-4 and IFN-γ expression pattern was described, instead, in vulvar LP. Evidence regarding IL-17 involvement remains contradictory: while protein-level analyses revealed significant elevations in both the epidermis and dermis [56], genomic studies found no significant dysregulation of Th17-axis genes (such as IL-17A, IL-17F, and IL-22R) or Th2-responses in LS [54].

The inflammatory response in LS is also attributed to a reduction in regulatory T-cell (Treg) activity, accompanied by lower levels of IL-10, without a parallel decrease in TGF-β levels [22,57]. IL-10 is an anti-inflammatory mediator that modulates immune responses, whereas TGF-β is essential for Treg differentiation and function [58,59].

The expression of Foxp3, a transcription factor of Treg cells, is significantly lower in vulvar LS compared to controls, suggesting involvement in immune tolerance breakdown and immune response against self-antigens [54,60,61]. Research has demonstrated a downregulation of Foxp3 expression and altered levels of the phenotypic marker CD127 in the peripheral blood of patients with lichen sclerosus. Notably, since the absolute count of CD4+ CD25+ Treg cells remains stable, these findings suggest that functional impairment, rather than numerical deficiency, of the Treg population may contribute to the loss of self-tolerance and subsequent autoimmune reactivity in LS [14,23,50,54].

MiR-155, a key microRNA in immune regulation, is significantly upregulated in LS lesions, promoting a Th1-dominant autoimmune response [54]. It enhances fibroblast proliferation by targeting FOXO3 and CDKN1B, contributing to sclerotic tissue formation characteristic of LS [62]. High miR-155 levels also reduce regulatory T-cell suppression, exacerbating inflammation in vulvar and male genital LS.

Overall clinical and experimental data indicate that the pathogenesis of LS is driven by a Th1-dominant inflammatory milieu and aberrant cytokine signaling exacerbated by functional impairment of the Treg population, leading to a breakdown in immune tolerance, which exacerbates tissue damage and perpetuates the cycle of disease progression.

Keratinocytes are fundamental orchestrators of the cutaneous immune response, mediating complex interactions within the skin microenvironment. Recent studies increasingly emphasize the contribution of epithelial dynamics, particularly keratinocyte stress and dysregulated epithelial–immune communication, to the inflammatory cascade in LS. Specifically, spatial and single-cell transcriptomics have highlighted keratinocytes as major functional components of vulvar lichen sclerosus pathogenesis [63], while observational data evaluating cytokine and chemokine profiles in cultured keratinocytes and fibroblasts further demonstrate their pro-inflammatory potential [64]. Rather than acting merely as passive targets of injury, stressed keratinocytes appear to actively modulate the microenvironment by releasing alarmins and pro-inflammatory mediators that recruit and activate infiltrating immune cells, thereby contributing to persistent local tissue dysfunction [1,2].

### 6.2. Fibrosis

A distinctive characteristic of the cutis involved in LS is the structural modification of the dermal compartment due to exacerbated deposition of collagen type I and III and loss of elastin fibers [65].

The extracellular matrix protein 1 (ECM1) plays a key role in LS pathogenesis. Several investigations reported ECM1 downregulation in LS skin [66,67]. However, impaired ECM1 function might also be explained by the presence of autoantibodies against this protein. Oyama and collaborators [68] demonstrated the presence of anti-ECM1 autoantibodies in 75% of patient sera affected by vulvar LS, and subsequent studies confirmed these findings also in male genital LS [69]. These seem to be LS-specific autoantibodies, as they were absent in healthy subjects and other sclerosing and/or autoimmune skin diseases, such as lupus, systemic sclerosis, and bullous pemphigoid.

Autoantibodies targeting ECM1 impair its regulatory function, leading to increased collagenase activity and triggering a cascade of pathological changes, such as dyskeratosis and thinning of the epidermis, homogenization of collagen fibers in the dermis, and the development of telangiectasias with increased vascular permeability, contributing to the progressive damage of the skin and sclerosis of the dermis that characterizes LS [1,70].

ECM1 is a multidomain glycoprotein primarily synthesized by basal layer keratinocytes and papillary dermal fibroblasts. It functions as a critical molecular scaffold, interacting with various structural proteins to maintain the mechanical integrity and architectural organization of the cutaneous extracellular matrix [19]. In the context of vulvar LS, reduced ECM1 expression is significantly associated with the depletion of elastic fibers; conversely, a negative correlation exists between ECM1 levels and collagen V content, suggesting a disrupted regulatory balance in lesional skin. This remodeling is further driven by TGF-β, a master regulator of fibrosis that induces fibroblast activation and myofibroblast differentiation. The resulting aberrant accumulation of ECM components contributes to the progressive tissue scarring and functional impairment characteristic of the disease [71].

Beyond its immunoregulatory role, elevated expression of TGF-β, its receptors, and matrix metalloproteinases (MMPs) characterizes LS tissue [1]. While immunohistochemical studies of pediatric phimosis and vulvar LS confirm increased TGF-β levels [72], its exact pathogenetic role remains debated. Interestingly, recent single-cell sequencing and spatial transcriptomics suggest an unexpected reduction in TGF-β signaling activation within lesional skin [73]. Given its central fibrogenic role, the dysregulation between latent and active TGF-β forms likely drives the disease. Like mechanisms in liver fibrosis [74], ECM1 alterations may disrupt extracellular matrix organization and modulate TGF-β activation, further exacerbating the characteristic fibrotic changes in LS.

In cases of LS-induced congenital phimosis, TGF-β and BMP2 are overexpressed, with their respective receptors acting as central regulators of fibrosis and tissue turnover [72]. Pathologically, anti-ECM1 autoantibodies abolish the regulatory inhibition of MMP9, thereby enhancing collagenase activity and disrupting the basement membrane zone [75].

Paradoxically, MMP9 hyperactivity can cleave and activate latent TGF-β, further stimulating collagen synthesis [51]. Beyond the accumulation of collagen types I and III, LS is characterized by abnormal collagen V deposition, upper dermal elastic fiber depletion, and diminished ECM1 expression within hyalinized blood vessels [67]. Additionally, galectin-7, a pro-apoptotic protein upregulated in the vulvar LS epidermis, contributes to disease progression by inhibiting keratinocyte viability, ultimately driving epidermal atrophy and stimulating fibroblast activity [76].

Galectin-7 could also act in a paracrine manner on fibroblasts, enhancing the transcription of type I and III collagen and downregulating the cell growth rate [77].

Hyperfunctional fibroblasts are central to the structural dermal remodeling observed during disease progression. In male LS, an enhanced crosstalk has been identified between fibroblasts and T cells via the collagen–CD44 axis, as well as between fibroblasts and keratinocytes through the APP-CD74 (Amyloid Precursor Protein-Cluster of Differentiation) signaling pathway [78]. This molecular dialog is implicated in the recruitment of immune infiltrates and the development of hyperkeratosis at the dermo-epidermal junction.

Furthermore, elevated CD44 expression may account for hyaluronate accumulation in LS skin [79]. However, diminished CD44 levels within the epidermis suggest a primary keratinocyte-driven alteration rather than a defect in the dermal fibroblasts, where sclerosis predominantly manifests [80].

Emerging evidence also highlights the role of factors secreted by both keratinocytes and fibroblasts, such as Dkk-1 (Dickkopf-1), GDF-15 (growth differentiation factor 15), IGFBP-2 (insulin-like growth factor-binding protein 2), and CHI3L1 (chitinase-3-like protein 1), suggesting that resident skin cells actively drive pathogenesis. Nevertheless, further investigations are required to fully elucidate the complex interactions between keratinocytes and fibroblasts in the development of vulvar LS [81].

### 6.3. Oxidative Stress

Oxidative stress, a critical driver in various autoimmune and malignant disorders, is increasingly recognized as a central mediator in the pathogenesis of LS. It likely functions as the mechanistic link among chronic inflammation, progressive tissue damage, autoimmunity, and the elevated risk of carcinogenesis.

Evidence of this process includes a marked increase in lipid peroxidation products within the basal epidermal layers, notably co-localizing with ECM1. Furthermore, the detection of oxidative DNA damage throughout LS biopsies suggests that the oxidative modification of lipids, DNA, and proteins contributes significantly to characteristic sclerosis, the breakdown of immune tolerance, and the potential for malignant transformation in LS.

Oxidative stress is thought to play a role in LS pathogenesis, as reactive oxygen species may contribute to tissue damage, autoimmunity, tumorigenesis, and dermal vasoconstriction.

In keratinocytes of chronic LS lesions, low levels of antioxidant enzymes, such as superoxide dismutase, and high levels of lipid peroxidation, oxidative DNA damage and protein oxidation [21,82] have been observed. Accumulation of oxidative DNA damage in LS may alter the expression of tumor suppressor proteins such as p27Kip1, potentially impairing cell-cycle regulation and contributing to an environment susceptible to neoplastic transformation [83].

Oxidative disequilibrium potentially creates new epitopes that can trigger autoimmune responses, also promoting the overexpression of wild-type p53 in basal keratinocytes, a compensatory mechanism aimed at counteracting the damaging effects of oxidative stress [11,51]. Additionally, due to the development of sclerotic vessels and poor oxygenation, the restriction of oxygen flow induces ischemic stress, intensifying the p53 overexpression [84].

## 7. Systemic Markers

Most studies have focused on lesional skin, while little is known about circulating cytokines. A study in 2005 investigated systemic immunological markers in LS patients, finding that while the absolute counts of circulating CD4+ and CD8+ T cells remained within normal ranges, there was a significant decrease in the proportion of CD4+ CD25+ regulatory T cells. Additionally, the study observed a reduction in Foxp3 expression and diminished levels of IL-10, suggesting that a systemic deficiency in suppressive immune mechanisms facilitates the loss of self-tolerance. These findings suggest that LS may not be merely a localized skin disease, but rather a condition associated with broader immune dysregulation that predisposes individuals to autoimmune reactivity [57]. Moreover, the frequent presence of anti-nuclear antibodies (ANA) positivity supports the systemic autoimmune etiology, but it is not a disease-specific marker [85]. According to data demonstrating a more frequent association with autoimmune disease in female LS patients compared to males (18.9% and 5.1% respectively) [18], similar gender differences have been published for ANA positivity (9.6% for females and 0.7% for males) [86].

The detection of circulating autoantibodies represents an additional possible marker. Anti-ECM1 antibodies are the most frequently detected, but their pathogenic role is still debated.

BP180 autoantibodies, targeting the basement membrane zone protein, are detected in some LS patients, particularly childhood vulvar cases (up to 44% by immunofluorescence/immunoblotting detection methods) [87]. These antibodies appear in ~30% of adult LS sera, alongside BP230 reactivity, but show no correlation with disease activity or pruritus [88].

## 8. Miscellaneous Other Proposed Triggering Factors

### 8.1. Trauma and Irritation

Mechanical trauma, including occlusion, friction, chronic scratching, and surgical interventions, alongside chronic irritation, is a reported trigger for LS lesions in both sexes [10]. In elderly women, factors such as urinary incontinence [89], multiparity, and deficient genital hygiene have been implicated in disease onset. Conversely, in male patients, chronic irritative penile micro-incontinence, anatomical abnormalities (e.g., hypospadias), and surgical procedures such as penile prosthesis implantation [90] act as predisposing factors. Furthermore, isolated reports have identified localized triggers such as insulin injections [91], influenza vaccinations [92], and intramuscular drug administration [93], suggesting a possible Koebner phenomenon in susceptible individuals.

### 8.2. Hormones

Analysis of the systemic endocrine profile in untreated women with vulvar LS revealed significant hormonal alterations, including elevated levels of circulating free testosterone alongside a marked reduction in dihydrotestosterone (DHT) and androstenedione compared to age-matched norms [94]. Furthermore, immunohistochemical investigations have demonstrated a progressive downregulation of nuclear androgen receptors (AR), as the disease transitions from early to advanced stages [95]. However, whether these hormonal and receptor changes represent primary etiopathogenetic drivers or secondary downstream effects remains to be established.

### 8.3. Infections

Evidence suggests that certain infections may act as catalysts for LS onset [96,97]. A 2025 study [98] identified prior diagnoses of human papillomavirus (HPV) and herpes simplex virus (HSV) as significant risk factors, noting that the incidence of LS appeared to decrease following HPV vaccination. The authors hypothesized that these viral agents might directly contribute to LS pathogenesis or, more likely, act as triggers for an autoimmune cascade through molecular mimicry or localized tissue stress.

Furthermore, the study revealed a higher prevalence of antecedent infections in LS patients, including gonorrhea, chlamydia, Lyme disease, and hepatitis B and C viruses. While other infectious correlations have been explored, their roles remain largely suggestive and lack definitive verification, necessitating further longitudinal research to confirm a causal link [14].

### 8.4. Medications

The literature contains sporadic reports linking the onset of LS and the administration of specific pharmacological agents. Imatinib mesylate caused LS in patients with chronic myelogenous leukemia and gastrointestinal stromal tumor [99,100]. In another case report, a 73-year-old man with epilepsy developed generalized LS [101] after 6 months of treatment with carbamazepine. Anogenital LS was also reported in adult patients with malignancy, treated with immunotherapy (pembrolizumab, nivolumab, or ipilimumab) [102,103,104]. Given the critical nature of these antineoplastic treatments, LS is managed similarly to other dermatological adverse events, to avoid the discontinuation of essential life-saving therapy [105]. Baldo et al. [106] investigated the impact of angiotensin-converting enzyme (ACE) inhibitors and beta-blockers in patients with vulvar lichen sclerosus. Their findings indicate that ACE inhibitors attenuate the inflammatory cellular infiltrate within the dermis. Conversely, beta-blockers were observed to reduce cyclic adenosine monophosphate (cAMP) levels, thereby stimulating keratinocyte proliferation and enhancing lymphocyte motility. These pharmacological interactions suggest that systemic medications may modulate the localized immune environment and epithelial dynamics in vulvar LS.

## 9. Treatments of Lichen Sclerosus: From Conventional to Regenerative Therapies

Therapeutic strategies for LS must prioritize the rapid relief of debilitating symptoms, such as pruritus, pain, and burning, to limit scarring, structural resorption, cutaneous atrophy, and potential malignant transformation. Moreover, management aims to decrease the frequency of flare-ups while maintaining and restoring patients’ quality of life. In addition to tailored personal hygiene and the avoidance of mechanical friction (Koebner phenomenon), current treatment modalities are classified into topical, systemic, and regenerative therapies (Table 1).

Among the principal topical therapies, the application of skin barrier emollients is recommended for their good tolerance and lack of safety concerns (unless allergies or irritants are present) [107]. Topical ultrapotent or potent corticosteroids, such as clobetasol propionate, represent the first-line therapy and gold standard of treatment for LS, exerting anti-inflammatory, anti-fibrotic, and anti-pruritic effects by switching off activated pro-inflammatory genes. Intralesional corticosteroids may be considered in refractory cases demonstrating a lack of response to topical therapy. Additionally, topical calcineurin inhibitors, such as pimecrolimus and tacrolimus, serve as a second-line therapeutic option, typically reserved for patients who are intolerant to or fail to respond to topical corticosteroids. These agents act by blocking the transcription of inflammatory cytokines, thereby reducing antigen presentation, T-cell activation, and the cellular components of non-humoral immunity implicated in the disease [108,109,110].

Furthermore, various emerging modalities, such as topical retinoids, hormonal preparations, ultraviolet (UV) phototherapy, photodynamic therapy, laser treatment, and cryotherapy, have recently been proposed. However, high-quality clinical evidence confirming their efficacy as non-standard or alternative treatments for LS therapies remains limited.

Various systemic therapies, including oral retinoids, methotrexate, hydroxychloroquine, and systemic corticosteroids (such as prednisone, methylprednisolone, prednisolone, and oral triamcinolone), have been used when standard topical treatments prove ineffective [111].

Refractory cases of genital LS have additionally been treated with Janus kinase (JAK) inhibitors, small-molecule drugs that block the JAK-STAT (Janus kinase–Signal Transducer and Activator of Transcription) signaling pathway and have documented efficacy across several immune-mediated inflammatory dermatoses. Early clinical evidence, primarily from single-arm trials and case series, suggests that oral JAK inhibitors such as baricitinib and abrocitinib represent promising therapeutic options for refractory genital LS [112,113]. These agents provide rapid symptomatic relief—specifically targeting pruritus—and significantly enhance quality of life within the first few weeks of administration, with benefits persisting throughout the treatment period. Furthermore, reflectance confocal microscopy and dermoscopy have documented a marked reduction in inflammatory infiltrate density, reflecting a potent anti-inflammatory action that may mitigate long-term post-inflammatory sclerosis. Recently, the identified dysregulation of the IL-17/IL-23 axis in vulvar LS, which is linked to disease chronicity and tissue atrophy, further supports the rationale for targeted JAK inhibition as an effective therapeutic strategy [114].

## 10. Surgical and Regenerative Therapies

Surgical intervention represents an additional option, primarily aimed at restoring physiological function or managing premalignant and malignant lesions.

In recent years, increasing attention has focused on regenerative medicine, including platelet-rich plasma (PRP), mesenchymal stem cell (MSC) graft therapies, primary adipose-derived stem cells (ADSCs), and their combined applications, particularly for managing symptomatic LS patients who fail canonical therapies [115].

PRP is a concentrate of autologous platelets and platelet-derived growth factors such as platelet-derived growth factor (PDGF), vascular endothelial growth factor (VEGF), epidermal growth factor (EGF), transforming growth factor-β (TGF-β) and fibroblast growth factor (FGF) that have a potent ability to counteract sclerosis and loss of dermal architecture in LS, stimulating fibroblast proliferation, angiogenesis, collagen and elastin synthesis and extracellular matrix remodeling. The application of PRP in genital lichen sclerosus has shown efficacy in promoting tissue regeneration and alleviating clinical symptoms through the release of growth factors that modulate the fibrotic microenvironment [116]. Clinical data indicate significant improvements in pruritus and dyspareunia, establishing PRP as a promising regenerative alternative for patients refractory to conventional corticosteroid therapy [115,117,118]. Nevertheless, large-scale randomized controlled trials remain essential to standardize injection protocols and evaluate long-term therapeutic durability.

ADSCs possess high regenerative capacity due to their abundance of stem cells, secretion of trophic factors, and modulation of the local immune response. Nevertheless, the adipose tissue-derived secretome may represent a preferred cell-free alternative owing to its rich composition of growth factors, lipids, and extracellular matrix components essential for cell proliferation and tissue remodeling [115]. It was also observed that stem cell-enriched fat grafting had a positive effect on LS patients, reducing fibrosis, pain, burning, dyspareunia and restoring anatomical and functional outcomes [119,120]. ADSCs have an immune-regulatory function towards CD8+ T cells, ROS scavenger activity, stimulating the cell’s endogenous antioxidant capacity and extracellular matrix remodeling capacity [121].

## 11. Conclusions

In conclusion, genital lichen sclerosus remains a complex, multifactorial disease requiring a proactive, multidisciplinary approach to prevent irreversible structural damage and mitigate the risk of malignant transformation. Although high-potency topical corticosteroids remain the gold standard first-line therapy, emerging options, such as JAK inhibitors and platelet-rich plasma, represent promising modalities for recalcitrant cases. Recent diagnostic advancements have enhanced non-invasive monitoring and disease staging, supporting the overarching goal of long-term clinical stabilization. Future research must prioritize randomized clinical trials and standardized therapeutic protocols to further refine personalized management strategies and optimize long-term patient outcomes.

## Figures and Tables

**Table 1:** Therapeutic modalities: established, emerging and experimental therapies for LS.

Therapy’s Category	Therapy/Modality	Mechanism of Action
Established	- Skin barrier emollients, baseline - Topical ultrapotent corticosteroids, first-line - Topical calcineurin inhibitors, second-line	Epithelial barrier restoration Anti-inflammatory and T-cell suppression Inhibition of T-cell activation (IL-2 blockade)
Emerging	- Topical retinoids - Hormonal preparations - Photodynamic therapy (PDT) - Cryotherapy - Systemic therapies (retinoids, methotrexate, hydroxychloroquine, corticosteroids) - J AK inhibitors (topical or systemic) - Surgical interventions	Keratinocyte differentiation and anti-fibrotic effect Epithelial trophism improvement ROS-mediated immunomodulation Localized tissue destruction and remodeling Systemic immunosuppression and inflammation control IFN-γ/Th1 pathway blockade Anatomical restructuring and release of scarring
Experimental	- Platelet-rich plasma (PRP) - Cell-based therapies (autologous adipose stem cell (stromal vascular fraction graft)	Growth factor release for tissue regeneration, tissue repair, stem cell paracrine function

## Data Availability

No new data were created or analyzed in this study. Data sharing is not applicable to this article.
